# Fabrication of clay soil/CuFe_2_O_4_ nanocomposite toward improving energy and shielding efficiency of buildings

**DOI:** 10.1038/s41598-021-00347-x

**Published:** 2021-10-21

**Authors:** Shabnam Keykavous-Amand, Reza Peymanfar

**Affiliations:** 1 Department of Architecture, Energy Institute of Higher Education, Saveh, Iran; 2 Department of Chemical Engineering, Energy Institute of Higher Education, Saveh, Iran

**Keywords:** Chemical engineering, Energy, Pollution remediation, Electronic materials, Magnetic materials, Optical materials, Light harvesting, Energy harvesting, Chemical engineering, Nanophotonics and plasmonics, Electronic properties and materials, Magnetic properties and materials, Nanoparticles, Structural properties, Ceramics, Composites, Environmental, health and safety issues, Microwave photonics, Nanoparticles

## Abstract

In this research, the energy and shielding efficiency of brick, fabricated by clay soil, as a practical building material was reinforced using CuFe_2_O_4_ nanoparticles. Initially, the nanoparticles were fabricated using the sol–gel method and then loaded in the brick matrix as a guest. The architected samples were characterized by X-ray powder diffraction (XRD), Fourier transform infrared (FTIR), diffuse reflection spectroscopy (DRS), field emission scanning electron microscopy (FE-SEM), High-resolution transmission electron microscopy (HRTEM), vibrating-sample magnetometer (VSM), differential scanning calorimetry (DSC) thermograms, and vector network analyzer (VNA) analyses. IR absorption of the tailored samples was monitored under an IR source using an IR thermometer. IR absorption and energy band gap attested that inserting the nanoparticles in brick medium led to the acceleration of a warming brick, desirable for energy efficiency in cold climates. It is worth noting that the brick/CuFe_2_O_4_ nanocomposite achieved a strong reflection loss (RL) of 58.54 dB and gained an efficient bandwidth as wide as 4.22 GHz (RL > 10 dB) with a thickness of 2.50 mm, meanwhile it shielded more than 58% of the electromagnetic waves at X-band by only a filler loading of 10 wt%. The microwave absorbing and shielding characteristics of the composite are mainly originated from conductive loss, electron hopping, natural and exchange resonance, relaxation loss, secondary fields, as well as eddy current loss. Interestingly, the shielding property of the nanocomposite was significantly generated from its absorbing features, reducing the secondary electromagnetic pollutions produced by the shielding materials applying the impedance mismatching mechanism.

## Introduction

Nowadays, the electromagnetic pollutions, emitted by the electronic devices developed for the artificial intelligence, 5G internet, etc. have exited the global concern in this era of communication^1,2^. Till date, diverse microwave absorbing and shielding materials have been architected against harmful electromagnetic pollution, improving military, industrial, and environmental applications. All in all, the permeability, permittivity, and impedance matching are the vital parameters dealing with the microwave absorbing and shielding characteristics^3–13^. It is worth noting that the conventional shielding materials benefit from the impedance mismatching augmenting the secondary pollution produced at the shielding structure. To offset this shortcoming, the impedance of electromagnetic shielding materials was tuned and they were tailored based on the absorbing characteristics. Diverse magnetic and dielectric structures comprising conductive polymers as well as oxide and sulphide structures have promoted the microwave characteristics. Among them, the nanostructures play the crucial role in microwave absorption, due to the considerable surface to volume ratio elevating the interfacial interactions and reinforcing interfacial and dipole polarizations^14–16^. The recent works have attested that the CuFe_2_O_4_ spinel nanoparticles have clarified the significant microwave features generated from their salient magnetic and dielectric characteristics. It should be noted that the fabricated microwave stealth materials are suspended in the thermoplastic and thermoset polymers to examine their microwave properties^5,17^. Over the last few years, diverse microwave absorbing and shielding media consisting silicone rubber, polyvinyl chloride, polystyrene, epoxy, polyacrylonitrile, polymethylmethacrylate, self-healing hydrogel, polyester, cement, concrete, polyurethane, polyurethane foam, and polyvinylidene fluoride have been applied, having their unique properties toward specific applications^18–20^. In this research, brick was chosen to architect the microwave absorbers. The applied matrix is a popular structure to construct buildings; moreover, it is an affordable and eco-friendly precursor with proper mechanical features for practical applications. In other words, the tailored nanocomposite based on the brick as the matrix has salient economic sustainability/industrial viability, augmenting the perspective of its cost-effectiveness for large-scale synthesis. On the other hand, the global warming has been the sleeping giant awaiting to irrupt originated from the increase of the greenhouse gases in the atmosphere. One of the major factors amplifying the greenhouse gases is the burning of fossil fuels to provide the required energy toward cooling or warming the buildings^21^.

This research follows two vital approaches: (1) enhancing the microwave absorbing and shielding characteristics of the brick as a building material using capable filler (2) diminishing the energy consumption in cold climates based on the optical characteristics of the brick/CuFe_2_O_4_ nanocomposite. CuFe_2_O_4_ spinel nanoparticles were prepared using a citrate gel method and applied as a guest to elevate the microwave feature of the brick. The heat resistance of spinel cupper ferrite introduces it as a promising candidate for loading in the brick, moreover, its eye-catching microwave and optical characteristics promote its importance.

## Experimental

### Materials

All of the precursor used to prepare the spinel structures including ammonia solution 25.0–30.0%, Fe(NO_3_)_3_·9H_2_O (99.0–101.0%), citric acid monohydrate (99.5–100.5%), and Cu(NO_3_)_2_·3H_2_O (≥ 99.5%) were purchased from Merck. The used sandy and clay soils were obtained from a local area in Tabriz-Iran.

### Preparation of CuFe_2_O_4_ nanostructures

The spinel structures were prepared based on the recent reports. Briefly, the nitrate salts of Cu and Fe were dissolved in deionized water, following that citric acid was added with a stoichiometric molar ratio of cationic metals/citric acid equals 3. Afterwards, the pH was adjusted about 8.5 using ammonia solution. Finally, the heat treatments were done at 300 and 850 °C for 4 h to prepare the nanostructures^5,17,22,23^.

### Fabrication of brick/CuFe_2_O_4_ nanocomposite

**Figure 1:**
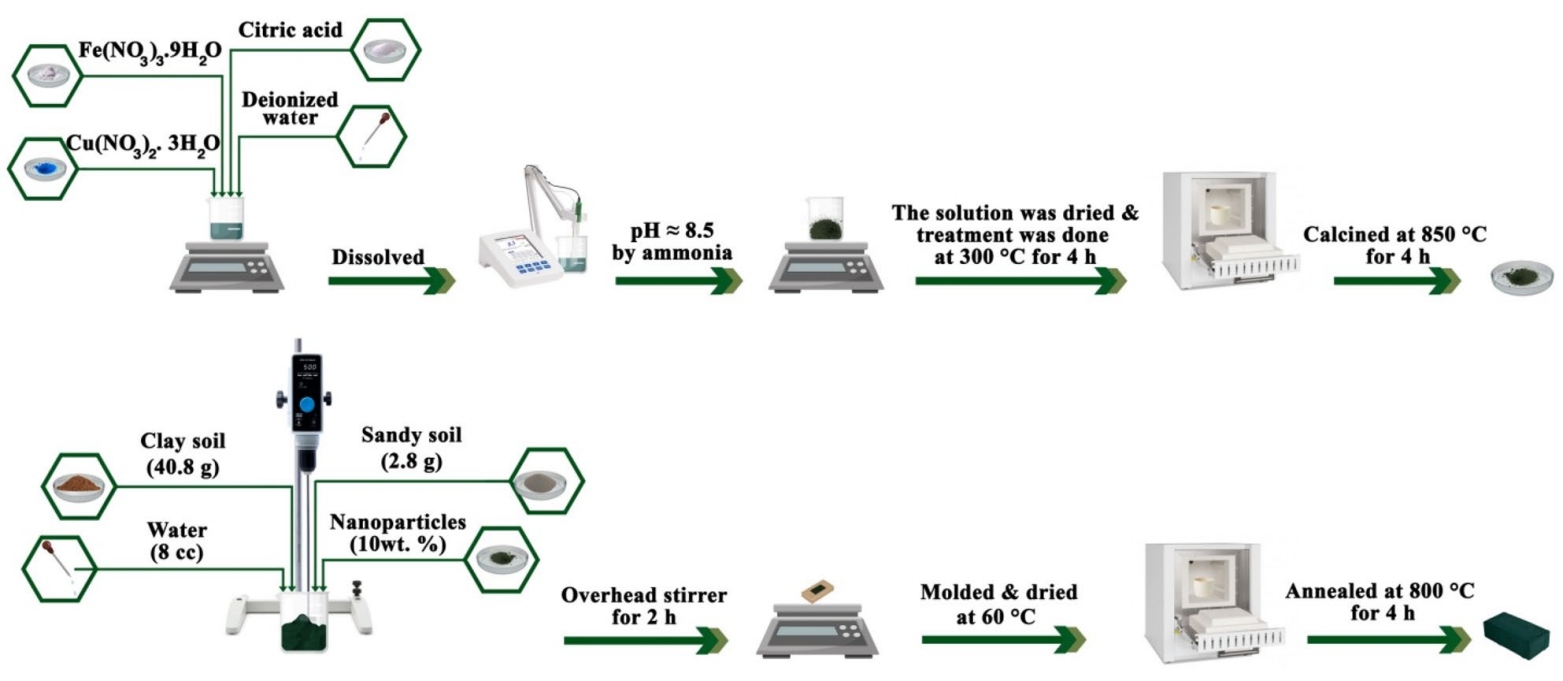
Schematic representation of the experimental procedures.

The initial paste was obtained by blending the clay soil (40.8 g) with deionized water (8 cc) and then the brick precursor was achieved by adding the sandy soil (2.8 g). Subsequently, the nanoparticles were loaded to the paste (10 wt%) and the substrates were blended by an overhead stirrer for 2 h. The aforementioned structure was molded in the rectangular shape (length = 22.86 mm, width = 10.16 mm, and thickness = 7.15 mm) and dried to investigate the optical, thermal, and microwave characteristics. Eventually, the brick/CuFe_2_O_4_ nanocomposite was obtained by annealing the composite at 800 °C for 4 h. Another sample without adding the nanoparticles was constructed, in the same conditions, to compare the results^21^. Figure 1 exposes a schematic representation of the experimental procedures applied to prepare brick/CuFe_2_O_4_ nanocomposite.

### Characterization

Chemical functional groups of the samples were evaluated by Shimadzu 8400 S meanwhile their crystal phases were revealed by Philips X'Pert MPD performing with a Co tube (λ = 1.78897 Å) at a range of 2θ = 10–70°, 40 mA, and 40 kV current. Morphologies of the samples were observed by micrographs obtained by Tescan Mira3. Furthermore, HRTEM analysis was done by FEI Tecnai G2 F20. Magnetic characteristics of the samples were evaluated using the IRI Kashan VSM at room temperature. Thermal behaviors of the structures were characterized using DSC and infrared thermometer from Tajhizat Sazan Pishtaz, Iran (TA-1) and Lutron TM-958. Optical and microwave features of the fabricated structures were examined using Shimadzu MPC-2200 and Agilent E8364A, respectively.

## Results and discussions

### Identification of chemical functional groups and crystal phases

**Figure 2:**
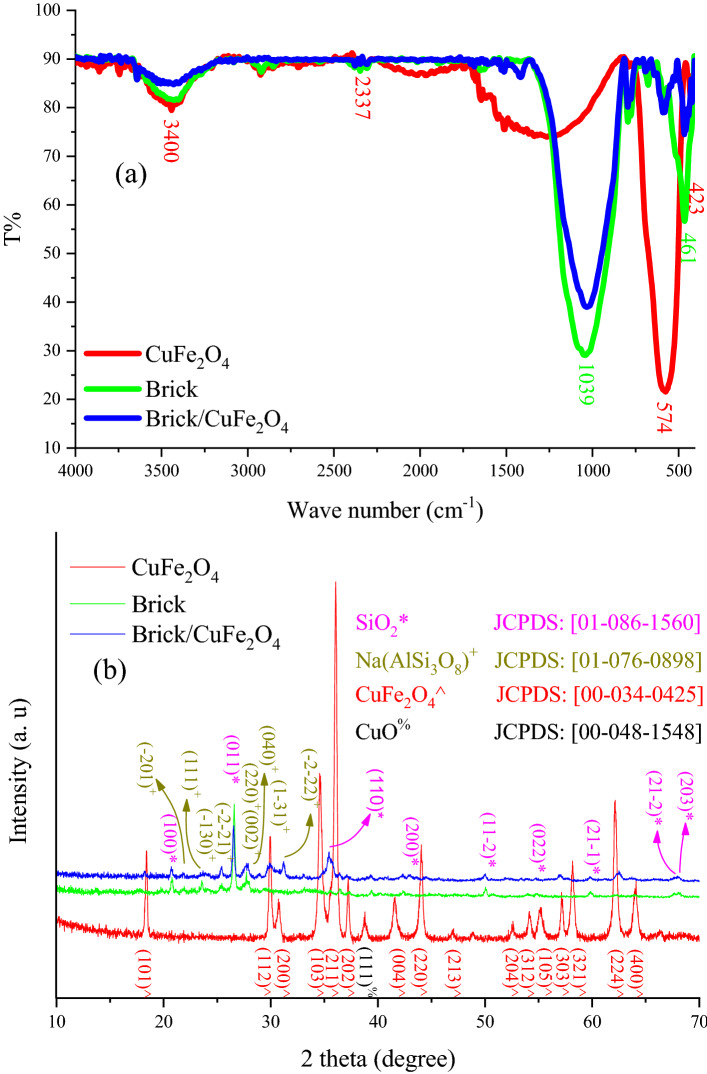
FTIR spectra (**a**) and XRD patterns (**b**) of CuFe_2_O_4_, brick, and brick/CuFe_2_O_4_.

The FTIR spectra and XRD patterns of CuFe_2_O_4_, brick, and brick/CuFe_2_O_4_ structures have been illustrated in Fig. 2. The achieved peaks at 423 and 574 cm^−1^ are attributed to the stretching vibration of Metal-O vibrations in octahedral and tetrahedral sites of CuFe_2_O_4_ nanoparticles^5,17^. On the other hand, the in-plane and out-of-plane bending vibrations of Si–O–Al and Si–O–Si were confirmed by the overlapped peak observed at 1039 cm^−1^ meanwhile the notch at 461 cm^−1^ attested to the stretching vibration of Si–O and Al–O existing in the brick^21,24^. Noticeably, the bump at 2331 cm^−1^ and the shallow band at 3400 cm^−1^ are ascribed to the adsorbed CO_2_ and H_2_O, respectively^25,26^. The XRD pattern of the brick showed that the dominant phases formed in brick are hexagonal SiO_2_ and anorthic Na(AlSi_3_O_8_) given by the JCPDS#: [01-086-1560] and [01-076-0898]. Additionally, the assigned peaks at CuFe_2_O_4_ pattern are suggesting that the tetragonal structures of CuFe_2_O_4_ nanoparticles have been synthesized corresponding to the JCPDS#: [00-034-0425] standard cart. Scherrer equation reported a crystallite size of 15.6 nm for the CuFe_2_O_4_ nanoparticles based on FWHM of (211) Brag reflection, meanwhile it was 16.2 nm for SiO_2_ obtained by characteristics of (011) crystal plane^5,17,21,27^. It is noteworthy that the assigned peaks at both spectrum and pattern of brick/CuFe_2_O_4_ nanocomposite, related to the presence of chemical functional groups and crystal phases of brick and CuFe_2_O_4_, demonstrate that the nanocomposite have been constructed and the experimental treatments have not any effect on their chemical and crystal structures. Noticeably, the XRD patterns of the clay and sandy soil as precursors were presented in Figure S1.

### FESEM micrographs

**Figure 3:**
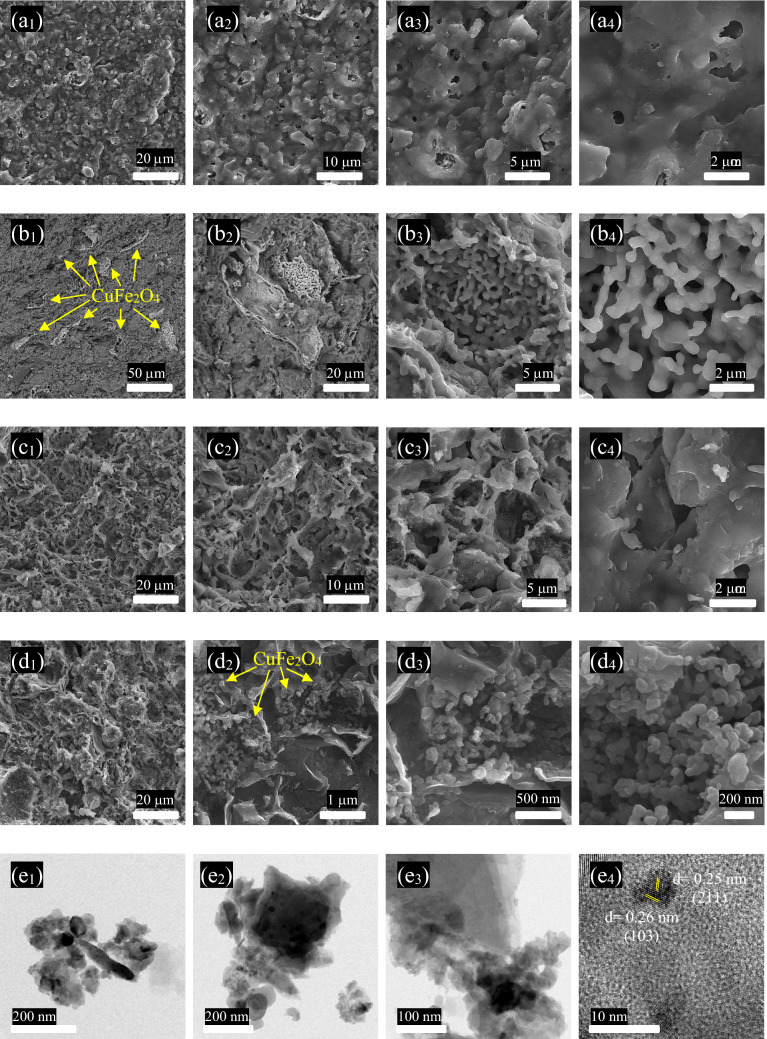
Surface images of brick (**a**_**1–4**_) and brick/CuFe_2_O_4_ (**b**_**1–4**_) and transect micrographs of brick (**c**_**1–4**_) and brick/CuFe_2_O_4_ (**d**_**1–4**_) as well as HRTEM images of brick/CuFe_2_O_4_.

Figure 3 displays surface micrographs of brick and brick/CuFe_2_O_4_ as well as transect images of brick and brick/CuFe_2_O_4_. Obviously, the integrated macroporous structure of the brick has been formed. It should be noted that the macroporous structure of brick augments the surface area to volume ratio of brick enhancing the interfacial interactions, desirable for relaxation loss. As revealed, the loaded CuFe_2_O_4_ nanoparticles with hierarchical morphology in the thickness range below 100 nm were evenly placed in the brick. As it can be seen, the surface and transect micrographs of the brick and brick/CuFe_2_O_4_ nanostructures are declaring that CuFe_2_O_4_ nanoparticles are loaded in the brick matrix which is in good agreement with the XRD patterns. More significantly, the results are clarifying that the morphology of the nanoparticles were maintained after their insertion in the brick medium. The different morphologies related to the nanoparticles and brick are clearly detectable in the FESEM micrographs^5,21,28^. Noticeably, the HRTEM images are confirming that the nanoparticles are properly implanted in the brick matrix.

### Optical performance

**Figure 4:**
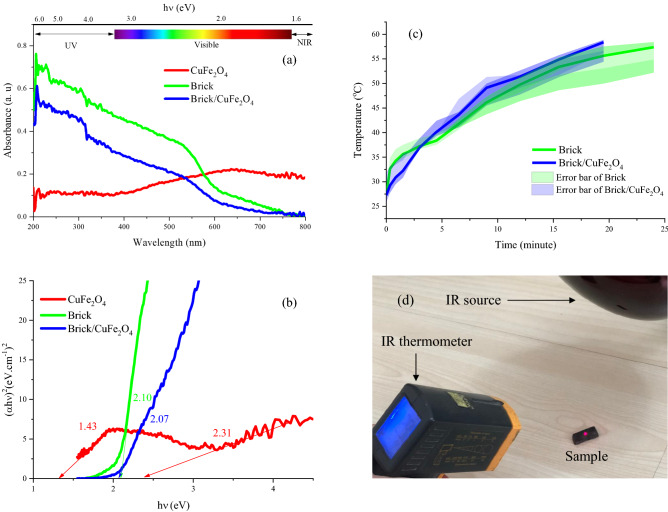
Optical characteristics comprising the light absorption at λ = 200–800 nm (**a**), energy band gaps (**b**), and IR energy absorption (**c**) of the samples as well as used setup to investigate IR absorption of samples (**d**).

UV–Vis light absorption is generally originated from the charge transitions from the valence band to conduction band. Figure 4 depicts the optical characteristics comprising light absorption at λ = 200–800 nm, energy band gaps, and IR energy absorption of the samples as well as used setup to investigate IR absorption of samples. It can be seen that the absorption edge of brick is λ = 620 nm dealing with its brown color. The obtained results illustrate that by diminishing frequency to the near IR, the light absorption of CuFe_2_O_4_ is amplified, generated from its intrinsic characteristics. Accordingly, inserting the nanoparticles in the brick matrix narrowed its energy band gap. The energy band gaps of prepared structures were examined by Kubelka–Munk theory^21,25,29^. Obviously, CuFe_2_O_4_ curve illustrated two energy band gaps (2.31 eV and 1.43 eV), corresponding to the energy gap related to CuFe_2_O_4_ and formed CuO, as confirmed by XRD pattern^30–33^. The wide light absorption around near IR can be associated with the local surface plasmon resonance and light scattering. It is well known that the size, shape, and defect of structures are the dominant parameters regulating the energy band gaps^14,16,34,35^. It is well known that the considerable portion of received sunlight is IR, increasing the earth temperature along a day. The potential of samples, related to their IR absorption, were investigated using a setup including the IR source, sample, and IR thermometer (Fig. 4d). Initially, the samples were placed under the IR source. After that, the samples were gradually warmed by the absorption of IR waves meanwhile the time was parallelly measured. The experimental process was repeated for three times to each sample, as indicated by the error bars. The ability of samples to convert electromagnetic waves in IR region to thermal was monitored until the samples achieved 58.5 °C. As revealed, brick/CuFe_2_O_4_ was quickly warmed obtaining 58.5 °C after 19 min and 30 s while it was extended to 24 min for Brick. Evidently, loading the nanoparticles in brick led to the acceleration of warming brick due to the optical features of CuFe_2_O_4_, as confirmed by the DRS results. The achieved results manifest that the presented scenario can be a promising approach toward promoting energy efficiency of buildings in cold climates by energy harvesting in IR and microwave region.

### Thermal features

**Figure 5:**
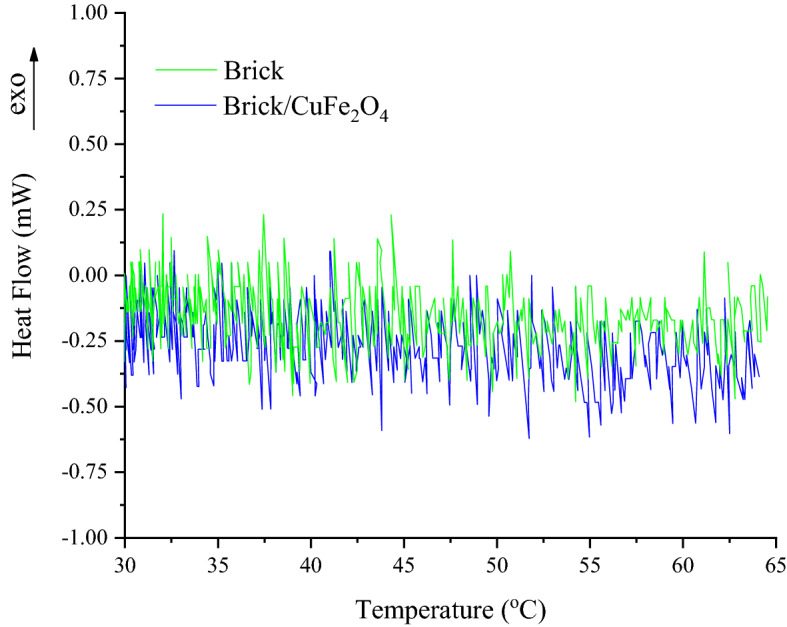
DSC thermograms of the brick and brick/CuFe_2_O_4_.

Thermal features of the brick and brick/CuFe_2_O_4_ have been illustrated in Fig. 5. The DSC thermograms clarified the thermal features of the samples from 30 to 65 °C. Noteworthy, inserting the nanoparticles augmented the needed energy for enhancing the temperature originated from the thermal capacitance of CuFe_2_O_4_.

### Magnetic hysteresis loops

**Figure 6:**
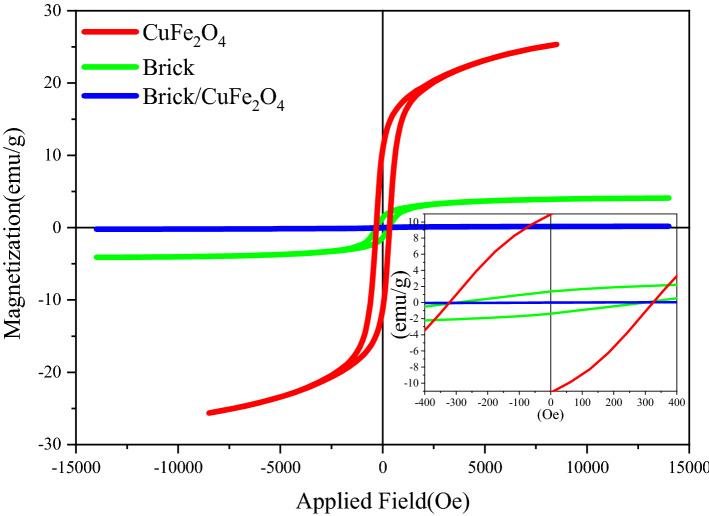
Hysteresis loops of CuFe_2_O_4_, brick, brick/CuFe_2_O_4_.

The applied field *versus* magnetization of CuFe_2_O_4_, brick, brick/CuFe_2_O_4_ is exhibited in Fig. 6. Additionally, the magnetic parameters including saturation magnetization (M_s_), remanent magnetization (M_r_), coercivity (H_c_) are summarized in Table 1. As reveled, brick do not show any considerable magnetic characteristics, on the other hand, it can be seen that by loading the nanoparticles in the non-magnetic matrix the magnetic parameters is diminished^15^. It is well known that the natural and exchange resonance play the vital role in microwave absorbing and shielding properties^36^.

**Table 1:** Magnetic characters of the samples.

Entry	Sample	M_s_ (emu/g)	M_r_ (emu/g)	H_c_ (Oe)
1	CuFe_2_O_4_	25.37	10.90	324.83
2	Brick	0.20	0.01	90.70
3	Brick/CuFe_2_O_4_	4.08	1.38	296.66

### Microwave characteristics

**Figure 7:**
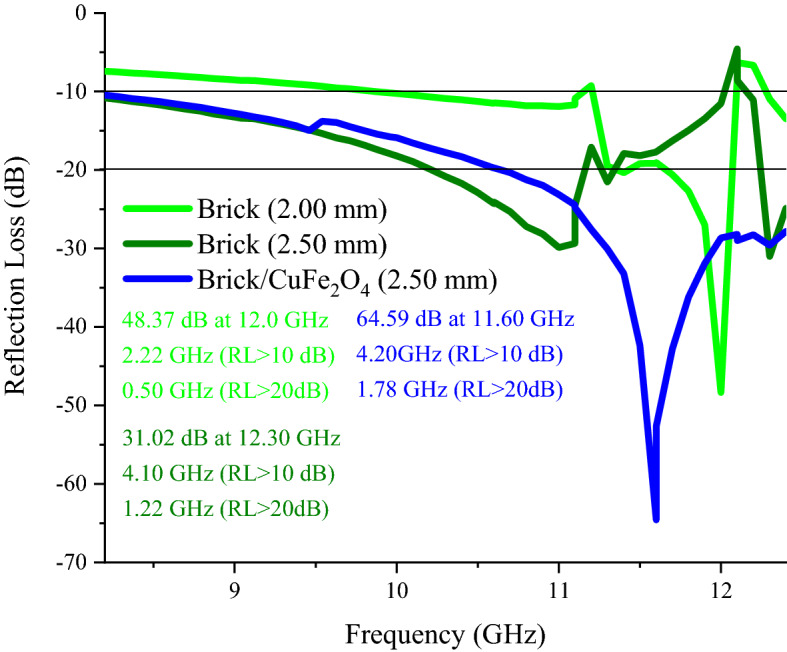
Microwave absorbing features of the samples.

**Figure 8:**
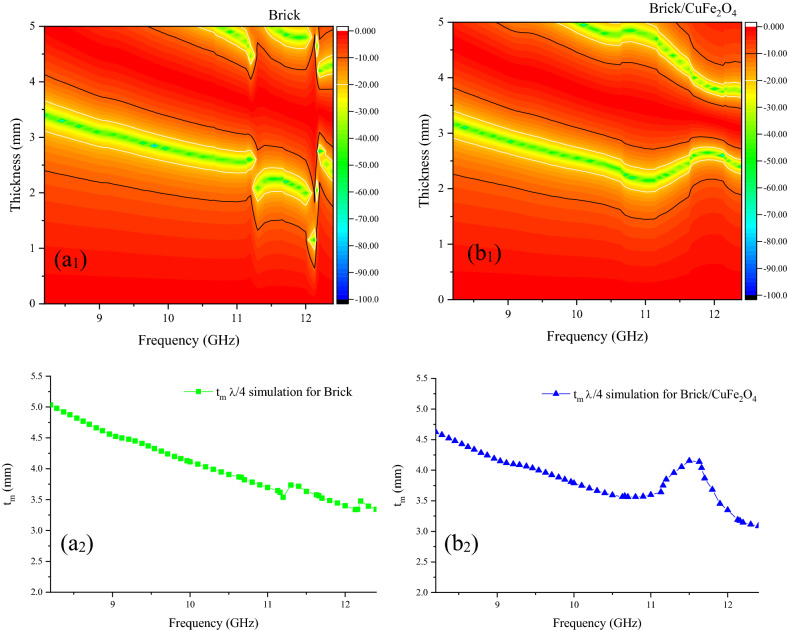
Frequency dependence of reflection loss and simulation of the matching thickness of the brick (**a**_**1, 2**_) and brick/CuFe_2_O_4_ nanocomposite (**b**_**1, 2**_) at X-band frequency.

**Figure 9:**
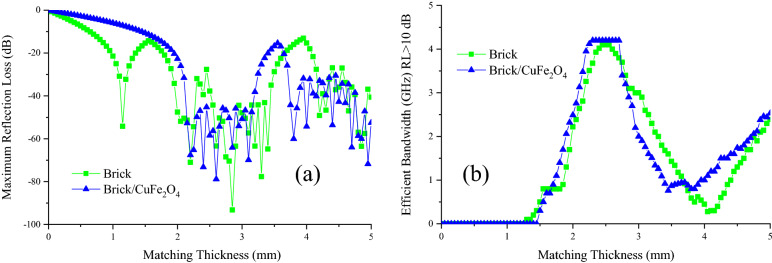
Matching thickness versus maximum reflection loss (**a**) and efficient bandwidth (**b**) of the samples.

Figures 7, 8, and S2 show microwave absorption and simulation of the matching thickness of the brick and brick/CuFe_2_O_4_ nanocomposite. The microwave absorbing properties were assessed based on the transmission line theory^37,38^. Accordingly, the permeability, permittivity, and impedance matching (Z) are the vital parameters paving the way for the microwave absorption. Besides, the simulation of the matching thickness was evaluated using quarter wavelength mechanism, declaring that the incident waves can be canceled by reversal waves from the reflector (180° out of phase) in which the thickness of absorber is odd numeral of λ/4 of penetrated wave^39–41^. The electrical conductivity and polarization play the crucial roles tailoring permittivity while natural and exchange resonance as well as eddy current effect tune permeability. Noteworthy, the electron hopping and charge circuits along the established loops as well as the aligned and ordered magnetic moments can develop induced secondary fields, declared by Lenz’s and Faraday's law ^42–48^. Figure 9 exhibits matching thickness versus maximum reflection loss and efficient bandwidth of the samples. Noteworthy, the brick/CuFe_2_O_4_ nanocomposite achieved strong RL of 58.54 dB and gained an efficient bandwidth as wide as 4.22 GHz (RL > 10 dB) with a thickness of 2.50 mm.

**Figure 10:**
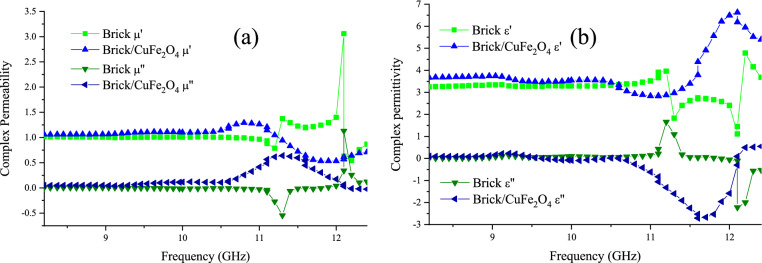
Frequency dependence of complex permeability (**a**) and permittivity (**b**) of the brick and brick/CuFe_2_O_4_ nanocomposite at 8.2–12.4 GHz.

Complex permittivity and permeability of the brick and the brick/CuFe_2_O_4_ nanocomposite at 8.2–12.4 GHz have been exposed in Fig. 10. As known, the real part of permeability and permittivity is derived from the storage of incident waves meanwhile the imaginary part of them is originated form attenuation. The results display that by loading the nanoparticles, the imaginary parts were totally promoted. There are diverse mechanisms that should be scrupulously dissected. The presence of the nanoparticles improved the imaginary part of the permeability owing to their natural and exchange resonance. On the other hand, the numerical values of the nanocomposite permittivity were augmented, associated with the enhanced dipole and interfacial polarization due to the enhanced grain boundaries attributed to the presence of guest. Furthermore, loading the nanoparticles elevate the electron hopping and conductive loss, known as major factors enhancing the imaginary parts of permittivity. It should be noted that the aligned magnetic dipoles under the alternating field establish the charge circuit in loops, developing secondary fields, metamaterial features, and negative parts.

**Figure 11:**
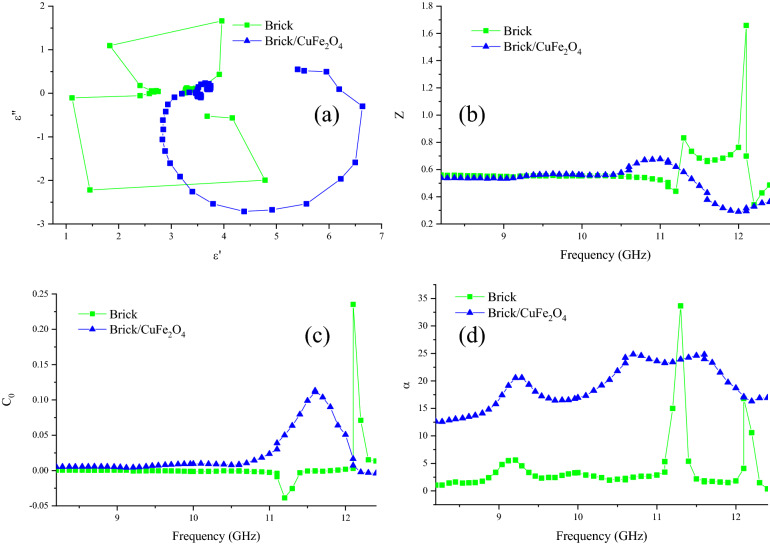
Cole–Cole plot (**a**), Z (**b**), C_0_ (**c**), and α (**d**) for the structures.

**Figure 12:**
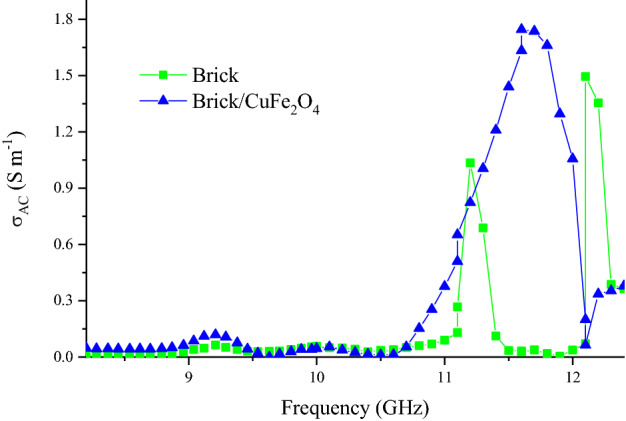
σ_AC_ for the architected samples.

Cole–Cole plot, Z, attenuation constant (α), eddy current loss (C_0_), skin depth (δ), and dissipation factor (tan δ) have been illustrated in Figs. 11 and S3. As indicated, the presence of nanoparticles enhanced the emerged semicircles in Cole–Cole plot attesting that the polarizability of composite is augmented, based on Debye relaxation theory^49^. It is noteworthy that each produced semicircle in Cole–Cole plot has the trade-off with one relaxation process. Z clarifies the potential of an absorber to percolating the incident waves from its threshold. The more closed Z to 1 declares the more propagated waves in the absorbing medium^50,51^. Obviously, Z is not the substantial factor of the obtained microwave characteristics. Interestingly, the eddy current loss plays the salient role bringing microwave absorption of brick. The more constant eddy current curve refers the more eddy current loss^52^. Subsequently, loading the nanoparticles diminished the eddy current loss after 10.5 GHz by augmenting the natural and exchange resonance. The amounts of α and tan δ demonstrate the susceptibility of an absorber for energy conversion^53^. It can be observed that by inserting the nanoparticles in the brick medium, the absorbing mechanisms consisting dipole and interfacial polarizations, conductive loss, natural and exchange resonances, and electron hopping are improved, promoting the permeability and permittivity, following that energy conversion of the composite. Figure 12 displays alternative conductivity (σ_AC_) for the architected samples. As reveled, the guest generally augmented the electrical conductivity of nanocomposite. The observed phenomenon is originated from the presence of nanoparticles in brick matrix augmenting imaginary part of permittivity; however, the guest has not any remarkable influence on δ.

**Figure 13:**
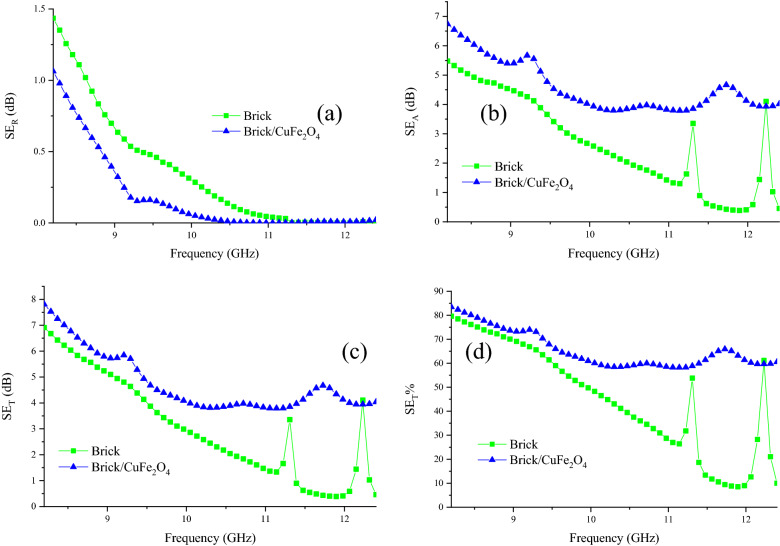
SE_R_ (**a**), SE_A_ (**b**), SE_T_ (**c**), and SE_T%_ (**d**) of the brick and brick/CuFe_2_O_4_ nanocomposite from 8.2 to 12.4 GHz.

**Figure 14:**
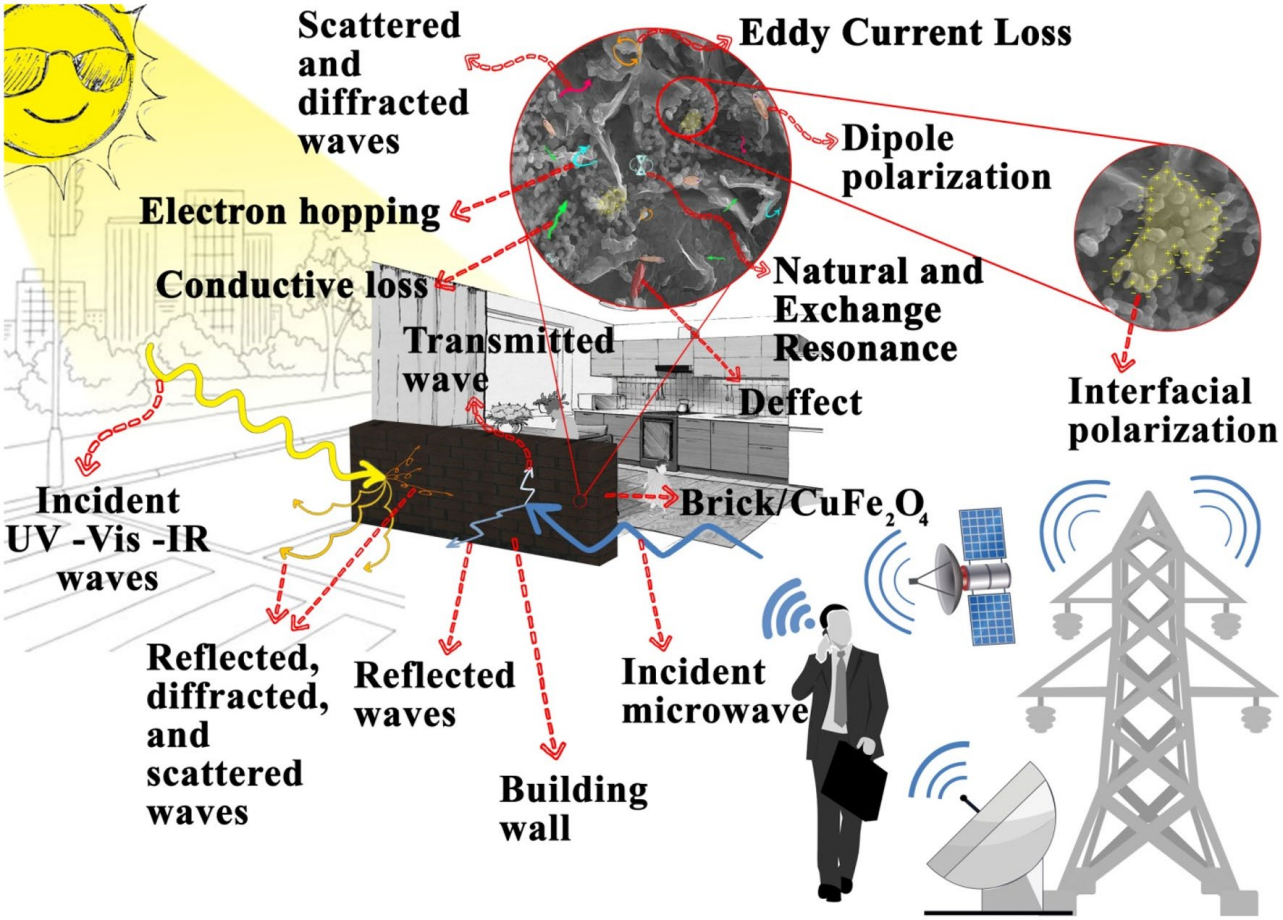
Schematic illustration of the microwave absorbing mechanisms existing in absorbing media.

Shielding characteristics of brick and brick/CuFe_2_O_4_ were explored using S parameters. SE of absorbance (SE_A_) and reflectance (SE_R_) are the vital factors bringing SE_T_. Interestingly, the results attested that the absorbance is the major parameter leading to the SE_T_ of the samples. Evidently, the nanocomposite has shielded more than 58% of the electromagnetic waves at X-band (Fig. 13). The SE_T_% is obtained from the following equation ${\text{SE}}_{{\text{T}}} \% = 100\left( {1 - 10^{{\left( { - \frac{{{\text{SE}}_{{\text{T}}} }}{10}} \right)}} } \right)$. It should be noted that the conventional shielding materials performing based on the reflectance can establish the secondary pollutions producing at their thresholds. To sum up, the attenuating feature of the samples, testified by their imaginary parts, is generated from the dipole and interfacial polarization, conductive loss, eddy current loss, natural and exchange resonance, electron hoping, and established secondary fields^15,19^. Obviously, with increasing frequency, total shielding effectiveness continuously decreases. The reason behind this phenomenon is originated from the reduction of the mentioned mechanisms as pioneer and dominant parameters, tuning shielding characteristics of the samples. Figure 14 depicts the mentioned microwave absorbing mechanisms existing in the absorbing media.

## Conclusion

This research shows the tip of an iceberg, illustrating the susceptibility of building materials as a matrix to reinforce energy and shielding efficiency. The employed analyses have testified that the structures were prepared and the nanoparticles were evenly dispersed in the brick medium. The obtained energy band gaps attested that the polarizability of the composite was enhanced, corresponding to the results achieved by monitoring IR absorption of the structures using IR source and thermometer. Interestingly, the results demonstrated that inserting the nanoparticles in the brick matrix improved the shielding and absorbing properties due to the augmented relaxation loss, conductive loss, electron hopping, natural and exchange resonance, secondary fields, as well as eddy current loss. Noteworthy, the shielding property of the nanocomposite was mainly originated from its absorbing features, diminishing the secondary electromagnetic pollutions produced by the shielding materials applying the impedance mismatching mechanism. The presented approach opens the new window toward improving energy and shielding efficiency in building materials, more significantly, can be a hotspot to architect the future researches.

## Supplementary Information

Supplementary Information.
