# Supplementary material for: Fabrication of clay soil/CuFe2O4 nanocomposite toward improving energy and shielding efficiency of buildings
Source: Sci Rep. 2021 Oct 21;11:20832. doi: 10.1038/s41598-021-00347-x (PMC8531380; doi:10.1038/s41598-021-00347-x)
Supplement: Supplementary file 1 — Supplementary Information. [file 41598_2021_347_MOESM1_ESM.docx]

**Supporting Information**

# Fabrication of clay soil/CuFe_2_O_4_ nanocomposite toward improving energy and shielding efficiency of buildings

Shabnam Keykavous-Amand ^a^, Reza Peymanfar^*b^

*a Department of Architecture, Energy Institute of Higher Education, Saveh, Iran*

*b Department of Chemical Engineering, Energy Institute of Higher Education,*

*Saveh, Iran*

**E-mail:* [*reza_peymanfar@alumni.iust.ac.ir*](mailto:reza_peymanfar@alumni.iust.ac.ir)

**1. Characterization**

**1. 1. XRD patterns**

**Figure S1.** XRD patterns of the clay and sandy soil as well as standard cards

**1. 2. Microwave absorbing features**

**Figure S2.** Microwave absorbing characteristics of the samples

(b)

(a)

**Figure S3.** δ (a) and tan δ (b) of the fabricated samples

**Table. S1.** Equations related to the microwave absorbing and shielding parameters [^1-14^](#_ENREF_1)

| **Title:** | **Equation/s:** |
| --- | --- |
| Transmission line theory | $R\left( \mathrm{dB} \right)=20Log\left\vert\frac{Z_{\mathrm{in}}-Z_{0}}{Z_{\mathrm{in}}+Z_{0}} \right\vert$, $Z_{\mathrm{in}}=\sqrt{\frac{\mu_{r}}{\varepsilon_{r}}}\tanh\left[ j\sqrt{\mu_{r}\varepsilon_{r}}f\left( \frac{2\pi}{c} \right)d \right]$,$Z_{0}=\sqrt{\frac{\mu_{0}}{\varepsilon_{0}}}$, $\varepsilon_{r}=\varepsilon'-j\varepsilon''$, and $\mu_{r}=\mu'-j\mu''$ |
| Quarter wavelength mechanism | $t_{m}=\frac{\mathrm{nc}}{4f_{m}\sqrt{\left\vert\varepsilon_{r} \right\vert\left\vert\mu_{r} \right\vert}}$ |
| Eddy current loss | $C_{0}={\mu''\left( \mu^{'} \right)}^{-2}f^{-1}$ |
| Debye relaxation theory | $\left( \varepsilon^{'}-\frac{\varepsilon_{s}+\varepsilon_{\infty}}{2} \right)^{2}+({\varepsilon^{''})}^{2}=\left( \frac{\varepsilon_{s}-\varepsilon_{\infty}}{2} \right)^{2}$ |
| Impedance matching | $Z=\frac{Z_{in}}{Z_{0}}$ $=\sqrt{\frac{\mu_{r}}{\varepsilon_{r}}}$ |
| Attenuation constant | $\alpha=\sqrt{\sqrt{{(\varepsilon_{r}''{\mu_{r}}^{''}-\varepsilon_{r}'{\mu_{r}}^{'})}^{2}+{(\varepsilon_{r}'{\mu_{r}}^{''}+\varepsilon_{r}''{\mu_{r}}^{'})}^{2}}+(\varepsilon_{r}''{\mu_{r}}^{''}-\varepsilon_{r}'{\mu_{r}}^{'})}\frac{\sqrt{2}f\pi}{c}$ |
| Dissipation factor | $\tan\delta=\frac{{\delta_{r}}^{''}}{{\delta_{r}}^{'}}$ , ${\delta_{r}}^{'}=\varepsilon_{r}'{\mu_{r}}^{'}-\varepsilon_{r}''{\mu_{r}}^{''}$, and ${\delta_{r}}^{''}=\varepsilon_{r}'{\mu_{r}}^{''}+\varepsilon_{r}''{\mu_{r}}^{'}$ |
| Skin depth | $\delta=\frac{1}{\sqrt{f\mu\pi\sigma_{\mathrm{AC}}}}$ |
| Alternative conductivity | $\sigma_{\mathrm{AC}}={\varepsilon''\omega\varepsilon}_{0}$,$\varepsilon_{0}=8.854*{10}^{-12}Fm^{-1}$, and$\omega=2\pi f$ |
| Electromagnetic interference SE | $\mathrm{SE}_{T}=\mathrm{SE}_{A}+\mathrm{SE}_{R}$, $\mathrm{SE}_{T}=-10\log\left\vert S_{12} \right\vert^{2}$,$\mathrm{SE}_{A}=-10\log(\frac{\left\vert S_{21} \right\vert^{2}}{{1-\left\vert S_{11} \right\vert}^{2}})$, $\mathrm{SE}_{R}=-10\log{(1-\left\vert S_{11} \right\vert}^{2})$, and $\mathrm{SE}_{T}\%=100(1-{10}^{\left( -\frac{\mathrm{SE}_{T}}{10} \right)})$ |

**Table. S2.** Definitions of the symbols

| **Symbol:** | **Definition:** | **Symbol:** | **Definition:** | **Symbol:** | **Definition:** |
| --- | --- | --- | --- | --- | --- |
| d | Thickness of absorber | c | Velocity of light in free space | Z_in_ | Input impedance |
| Z_0_ | Free space impedance | f | Frequency | n | Odd number |
| μ′ | Real part of permeability | μ″ | Imaginary part of permeability | t_m_ | Matching thickness |
| ε′ | Real part of permittivity | ε″ | Imaginary part of permittivity | f_m_ | Matching frequency |
| ε_∞_ | Permittivity at the infinite frequency | ε_s_ | Static permittivity | ε_0_ | Permittivity constant |
| ω | Angular frequency |  |  |  |  |

**References**

1 Zhang, S., Cao, Q., Zhang, M. & Shi, X. Effects of Sr2+ or Sm3+ doping on electromagnetic and microwave absorption performance of LaMnO3. *Journal of Applied Physics* **113**, 074903 (2013).

2 Qin, F. & Brosseau, C. A review and analysis of microwave absorption in polymer composites filled with carbonaceous particles. *Journal of applied physics* **111**, 4 (2012).

3 Peymanfar, R. & Azadi, F. Preparation and identification of bare and capped CuFe2O4 nanoparticles using organic template and investigation of the size, magnetism, and polarization on their microwave characteristics. *Nano-Structures & Nano-Objects* **17**, 112-122 (2019).

4 Zhang, X.-J. *et al.* Enhanced microwave absorption property of reduced graphene oxide (RGO)-MnFe2O4 nanocomposites and polyvinylidene fluoride. *ACS applied materials & interfaces* **6**, 7471-7478 (2014).

5 Peymanfar, R., Norouzi, F. & Javanshir, S. A novel approach to prepare one-pot Fe/PPy nanocomposite and evaluation of its microwave, magnetic, and optical performance. *Materials Research Express* **6**, 035024 (2018).

6 Moitra, D. *et al.* Synthesis and Microwave Absorption Properties of BiFeO3 Nanowire-RGO Nanocomposite and First-Principles Calculations for Insight of Electromagnetic Properties and Electronic Structures. *The Journal of Physical Chemistry C* **121**, 21290-21304 (2017).

7 Peymanfar, R., Norouzi, F. & Javanshir, S. A novel approach to prepare one-pot Fe/PPy nanocomposite and evaluation of its microwave, magnetic, and optical performance. *Materials Research Express* (2018).

8 Du, M. *et al.* Design of efficient microwave absorbers based on multi-layered polyaniline nanofibers and polyaniline nanofibers/Li0. 35Zn0. 3Fe2. 35O4 nanocomposite. *Synthetic Metals* **223**, 49-57 (2017).

9 Shu, R. *et al.* Facile preparation and microwave absorption properties of RGO/MWCNTs/ZnFe2O4 hybrid nanocomposites. *Journal of Alloys and Compounds* **743**, 163-174 (2018).

10 Almasi-Kashi, M., Mokarian, M. H. & Alikhanzadeh-Arani, S. Improvement of the microwave absorption properties in FeNi/PANI nanocomposites fabricated with different structures. *Journal of Alloys and Compounds* **742**, 413-420 (2018).

11 Bora, P. J., Azeem, I., Vinoy, K., Ramamurthy, P. C. & Madras, G. Morphology controllable microwave absorption property of polyvinylbutyral (PVB)-MnO2 nanocomposites. *Composites Part B: Engineering* **132**, 188-196 (2018).

12 Wang, Y. *et al.* Fabrication and enhanced electromagnetic wave absorption properties of sandwich-like graphene@ NiO@ PANI decorated with Ag particles. *Synthetic Metals* **229**, 82-88 (2017).

13 Weir, W. B. Automatic measurement of complex dielectric constant and permeability at microwave frequencies. *Proceedings of the IEEE* **62**, 33-36 (1974).

14 Peymanfar, R. & Rahmanisaghieh, M. Preparation of neat and capped BaFe2O4 nanoparticles and investigation of morphology, magnetic, and polarization effects on its microwave and optical performance. *Materials Research Express* **5**, 105012 (2018).
